# Role of cochlear reserve in adults with cochlear implants following post-lingual hearing loss

**DOI:** 10.1007/s00405-022-07558-6

**Published:** 2022-08-10

**Authors:** Kruthika Thangavelu, Markus Nitzge, Rainer M. Weiß, Jochen Mueller-Mazzotta, Boris A. Stuck, Katrin Reimann

**Affiliations:** Department of Otorhinolaryngology, Head and Neck Surgery, University Hospital Marburg, Philipps-Universität Marburg, Baldingerstrasse, 35043 Marburg, Germany

**Keywords:** Cochlear implant, Cochlear reserve, Tone hearing, Speech understanding, Speech perception gap

## Abstract

**Introduction:**

Pre-operative assessments before cochlear implantation (CI) includes the examination of both tone hearing, and the level of the cochlear reserve indicated by speech understanding. The goal of this study was to explore the predictive influence of tone hearing and cochlear reserve in CI.

**Methods:**

We did a retrospective cohort study, which included adult patients who had undergone CI between January 2012 and December 2019 in a tertiary care center. The pre-operative tone hearing, unaided maximum monosyllabic word recognition score (WRSmax), aided hearing gain, aided monosyllabic word recognition score at 65 dB (WRS65(HA)), and speech perception gap (SPG) were measured. The duration of unaided hearing loss (UHL) was also assessed. These variables were compared with post-operative monosyllabic word recognition score after CI at 65 dB (WRS65(CI)).

**Results:**

103 patients and 128 ears were included in this study. Regardless of tone hearing, patients with better pre-operative WRSmax and WRS65(HA) performed better post-operatively. WRSmax was found to be the most important factor that was statistically significantly associated with WRS65(CI). SPG was statistically significantly associated with WRSmax and SPG ≥ 20% group performed better post-operatively. Any duration of unaided hearing loss was statistically significantly inversely associated with WRSmax above 0%.

**Conclusion:**

Cochlear reserve represented by WRSmax may play the most important role as a predictive factor in outcomes after CI. SPG should be considered for indicating CI in patients, when WRS65(HA) does not reach WRSmax. Early rehabilitation with hearing aids and duration of hearing aid usage might play an important role in preserving cochlear reserve in adults.

## Introduction

Hearing loss affects more than 70% of the population aged 60 years and above worldwide and thus poses a significant threat to the quality of life and cognitive outcomes, particularly as life expectancy increases [10, 27]. Hearing rehabilitation with hearing aids is the most common treatment in this scenario. However, when best-fitting hearing aids fail to provide adequate benefit, cochlear implantation (CI) can improve hearing and thus quality of life significantly [4] in the affected population.

Recent technological advances of various components of the implant systems and improvements in surgical techniques have resulted in massive changes in candidacy criteria for CI in the last years. During the initial years of CI, only patients who were functionally profoundly deaf and had no speech perception with best-fitting hearing aids were considered as candidates for a CI. In recent years, the audiological indication criteria have become increasingly wider [9, 13].

In today’s context, many patients still have enough residual hearing and yet receive CI, since they do not benefit from hearing aids. Particularly for these patients, the individual prognosis of post-operative speech perception with respect to the preoperative assessment is an absolute clinical necessity. Patients need to be counselled accordingly before CI, since there is always the risk of impaired residual hearing [17, 18].

The pre-operative assessment before CI in patients includes the examination of both tone hearing, as well as the level of cochlear reserve, as indicated by speech understanding. Although it may seem intuitive that patients with better pre-operative tone hearing and a better pre-operative speech understanding should be associated with an improved speech comprehension after treatment with CI, viewpoints among otologists regarding the significance of each pre-operative parameter vary and the definitive identification of predictive factors remains elusive [24].

In daily clinical practice, we often experience that when patients with bilateral severe to profound sensorineural hearing loss and bad speech understanding receive CI on one side, the post-operative speech understanding seems to vary widely, with an outcome of the hearing rehabilitation ranging from absolutely unsatisfying to quite satisfactory [19]. This variation in outcome among patients with unilateral CI treatment and severe to profound contralateral sensorineural hearing loss has not been studied thoroughly yet. Here, the role of the duration of deafness and the duration of usage of hearing aids prior to CI treatment could also play a further crucial role.

The goal of this study was to explore the predictive influence of tone hearing, as well the influence of the cochlear reserve, indicated by pre-operative speech understanding, on speech comprehension after hearing rehabilitation. Included were patients with unilateral CI and contralateral severe to profound sensorineural hearing loss. Further, the study aims to explore the role of the duration of deafness and the duration of usage of hearing aids prior to CI for hearing outcome.

## Methods

### Study population

After obtaining approval of ethics committee of the medical faculty at the University (114/21), audiometric data from CI recipients were retrospectively queried using the electronic medical record and the CI registry. This was a retrospective cohort study which included patients who received CI from January 2012 to December 2019 at a tertiary care and cochlear implant center in Germany. The inclusion criteria were (1) Adults 18 or older of age, (2) Contralateral ear showed at least moderate or severe to profound hearing loss, (3) German speaker and (4) Post-lingual hearing loss. The exclusion criteria were (1) No cognitive disabilities, (2) No cochlear malformations, (3) No left corner hearing curve that is less than 40 dB hearing threshold across low frequencies and severe to profound hearing loss at high frequencies.

### Audiological tests

In the German speaking countries, monosyllabic and sentence tests are routinely used to evaluate patients with hearing aid and patients who receive CI. The Freiburg monosyllabic test is universally used in Germany, and it is conducted with headphones within the standardized speech audiogram as well as in the free field situation with hearing aids or with CI. This yields information about speech intelligibility at conversation levels and at the maximum frequency possible close to discomfort level.

In our center, the usual pre-operative audiological tests include the unaided pure ton audiogram and the aided hearing gain. The pure tone audiogram without hearing aid was measured and an average of the decibels measured at 500 Hz, 1, 2 und 4 kHz was taken as PTA4 (pure ton average of four frequencies). Similarly, the hearing again with hearing aid was measured and an average of the gains measured at 500 Hz, 1, 2 und 4 kHz was taken as FNA4 (fresh noise average of four frequencies). Then the unaided maximum recognition score (WRSmax) for phonemically balanced monosyllabic words was measured near the discomfort level using air-conduction headphones, which was usually at 110 decibels. Following WRSmax, the score for recognition of phonemically balanced monosyllabic words at conversation level of 65 dB with hearing aid was measured and referred to as WRS65(HA). WRS65(HA) was usually measured in free field in a non-echoic booth at 65 dB. The contralateral ear was always masked appropriately with wideband noise also presented using headphones. It was ensured that the hearing aid provided adequate amplification to ensure best aided hearing possibly achieved in the given patient. The speech perception gap (SPG) was measured for each ear from subtracting WRS65(HA) from WRSmax. A SPG equal to or greater than 20% was considered as a ‘high SPG’ based on prior literature and the cohort was grouped accordingly for further analysis [20]. The post-operative results after CI were also measured using the Freiburg phonemically balanced monosyllabic words test at conversation level of 65 dB (WRS65(CI)). The WRS65(CI) measured 1 year after CI activation was extracted from the CI registry. The contralateral ear was again masked appropriately with wideband noise also presented using headphones.

### Grouping of the cohort

PTA4 and WRSmax were plotted across *y*- and *x*-axis, respectively, and the cohort was divided into four groups (A, B, C and D). The cut-off for PTA4 was taken at 80 dB to differentiate moderate and severe hearing loss from profound hearing loss. A cut-off value for WRSmax was taken at 50% to differentiate relatively better speech perception from bad speech perception, since 50% was originally used as the cut-off value for CI indication. Following this, FNA4 and the WRS65(HA) were plotted across *y*- and *x*-axis, respectively, and the cohort was again divided into four groups (A1, B1, C1 and D1). The cut-off for FNA4 was 60 dB to differentiate relatively better hearing gain with hearing aid from lesser gain with hearing aid. The cut-off for WRS65(HA) was taken at 50%, since a speech perception above 50% was considered too good for CI until recently.

Based on the WRSmax for each ear the cohort was again divided into two groups: (1) ears with 0% WRSmax were designated as WRSmax0 and (2) ears with more than 0% WRSmax were designated as WRSmax > 0.

### Duration of hearing loss and hearing aid usage

An approximate duration of hearing loss and duration of hearing aid usage in months were extracted from the patient files. The duration of hearing aid usage was subtracted from the duration of hearing loss and the results were taken as the duration of unaided hearing loss (UHL) in months. UHL was then categorized into two groups: group 1 with zero values where in it was assumed that the ear had very minimal unaided hearing loss in months (UHL0) and group 2 where in the ear with hearing loss for any duration and was not supported with the help of a hearing aid during this time (UHL > 0). Group 1 with no UHL does not necessarily mean that the patients with any hearing loss immediately received hearing aid, since this variable was taken retrospectively, where patients were asked the question “since how long do you suffer from debilitating hearing loss and since how long do you use hearing aid?”.

### Statistical analysis

Pre-operative speech audiometry results, obtained using high power hearing aids, were compared with post-operative speech audiometry results using two sample *t* test and chi square tests. The post-operative speech audiometry results were compared among the three groups using Mann–Whitney test. The characteristics of the three distinct groups were compared using Chi-square test. Univariate linear regression was used to identify the possible associations between preoperative audiological tests, pre-operative duration of deafness and duration of hearing aid usage and post-operative outcome. Finally, correlation between pre-operative duration of deafness in years and the post-operative outcome were assessed using Spearman’s correlation coefficient. Group differences were considered significant if *p* value was less than 0.05. All analyses were performed using Stata 14.0 (StataCorp. 2014. *Stata Statistical Software: Release 14*. College Station, TX: StataCorp LP).

## Results

From January 2012 to December 2019, 471 patients received cochlear implantation in the tertiary care center. From these, 103 adult patients fulfilled the inclusion criteria. Of these, 25 patients received bilateral CI.

### Descriptive

The study population included 55 females and 44 males with the mean age of 63 ± 7 years. We analyzed each ear as an entity and thus 128 ears were included. The contralateral ear showed moderate or severe to profound hearing loss. The mean PTA4 was 93.7 ± 17 decibels (min: 50; max: 120). The mean FNA4 was 61.3 ± 18 decibels (min: 28; max: 102). The mean unaided WRSmax was 28 ± 28.8% (min 0; max 90) and the mean WRS65(HA) was 23 ± 20% (min:0; max 60). The overall SPG for the cohort was 6.3% (min: -35; max: 60).

### Influence of unaided tone hearing and unaided speech understanding

**Fig. 1:**
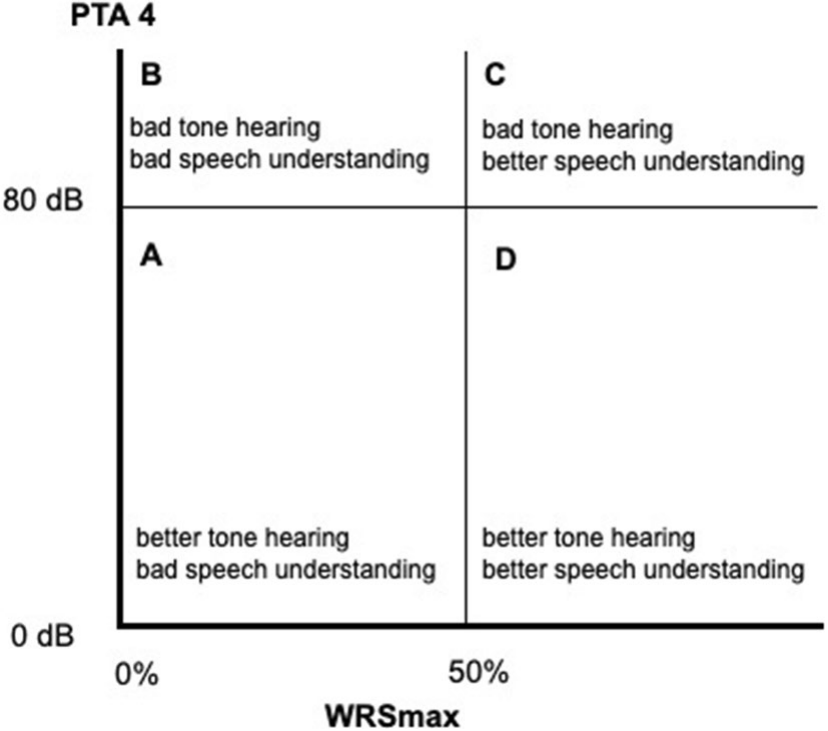
Groups formed based on PTA 4 and WRSmax plotted against each other

**Graph 1:**
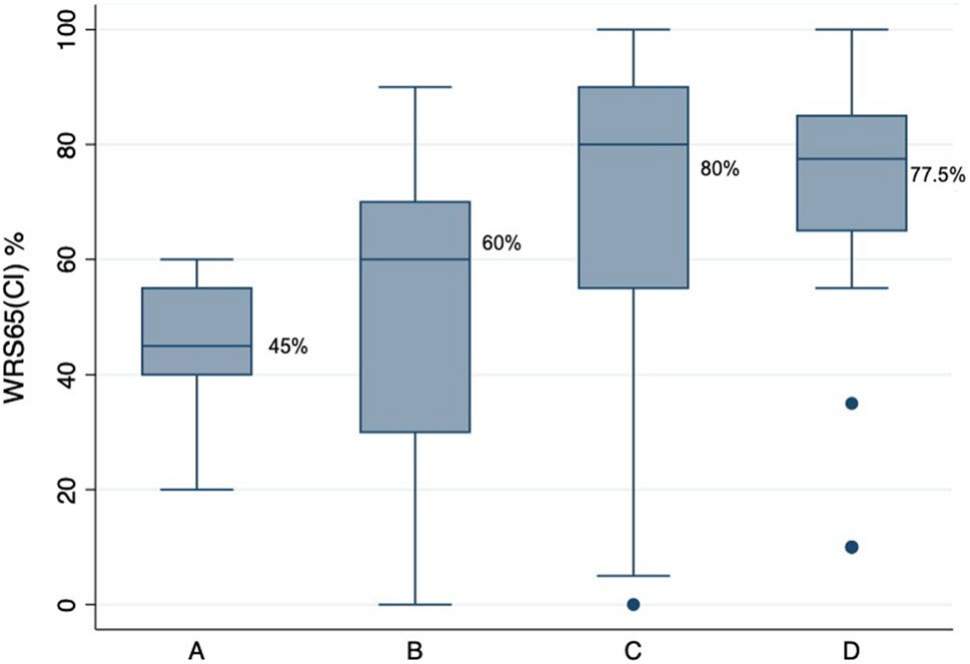
Boxplot showing median post-operative monosyllabic word recognition score at conversation level (65 dB) for every group based on Fig. 1

The four groups (A, B, C and D) and the post-CI results for these groups are shown in Table 1. The WRS65(CI) across groups showed that groups C and D with equal to or more than 50% WRSmax had a statistically significantly better post-operative speech understanding when compared to groups A and B, which had a WRSmax of less than 50% (Fig. 1, Graph 1). Thus, patients with unaided better speech understanding performed better regardless of unaided pure tone audiogram.

**Table 1:** Post-CI results for the cohort based on PTA4 and WRSmax

Groups	No. of ears (*N*)	Median WRS65(CI) (%)	Mean WRS65(CI) ± Standard deviation (%)
A. ≤ 80 dB and < 50%	10	45	46 ± 12
B. > 80 dB and < 50%	73	60	51 ± 27
C. > 80 dB and ≥ 50%	23	80	68.7 ± 29
D. ≤ 80 dB and ≥ 50%	22	77.5	68.4 ± 28

*T* test difference in mean between groups: C/A Diff 22.7 (95%CI 3.2–42.2) *t* statistic 2.371, *p* value 0.0241. C/B Diff 17.7 (95%CI 4.7–30.7) t statistic 2.694, *p* value 0.0084. D/B Diff 17.4 (95%CI 4.1–30.7) *t* statistic 2.605, *p* value 0.0107. D/A Diff 22.4 (95%CI 2.8–41.9) *t* statistic 2.336, *p* value 0.0263

### Influence of aided tone hearing gain and aided speech understanding

**Fig. 2:**
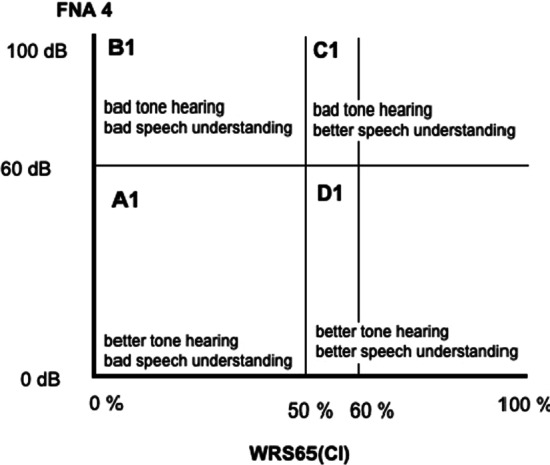
Groups formed based on FNA 4 and WRS65(CI) plotted against each other

**Graph 2:**
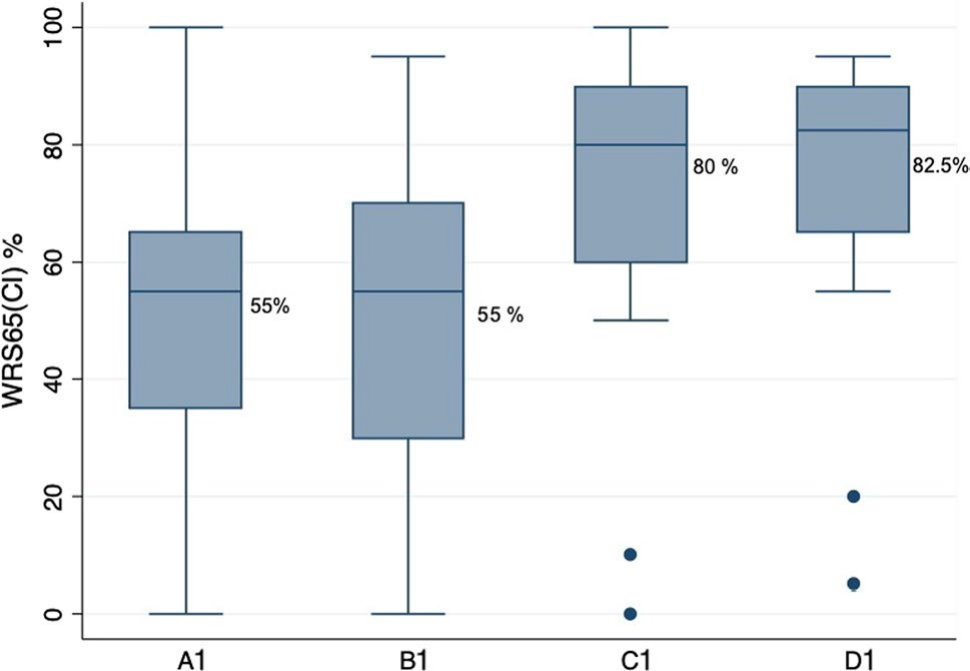
Boxplot showing median post-operative monosyllabic word recognition score at conversation level (65 dB) for every group based on Fig. 2

Similarly, the other four groups (A1, B1, C1 and D1) and the post-CI results are shown in Table 2. The WRS65(CI) across groups showed that the groups C1 and D1 with relatively better WRS65(HA) that is between 50 and 60% had a statistically significantly better post-operative speech understanding when compared with groups A1 and B1 which had less than 50% WRS65(HA) (Fig. 2, Graph 2). Thus patients with better-aided speech understanding performed better post-operatively regardless of their aided hearing gain in tone hearing.

**Table 2:** Post-CI results for the cohort based on FNA4 and WRS65(HA):

Groups	No. of ears (*N*)	Median WRS65(CI)(%)	Mean WRS65(CI) ± standard deviation (%)
A1. ≤ 60 dB and < 50%	39	55	51 ± 25
B1. > 60 dB and < 50%	55	55	51.5 ± 28
C1. > 60 dB und 50–60%	14	80	68.9 ± 30
D1. ≤ 60 dB und 50–60%	20	82.5	74 ± 24

*T* test difference in mean between groups: C/A Diff 17.9 (95%CI 1.4–34.4) *t* statistic 2.179, *p* value 0.0340. C/B Diff 17.4 (95%CI 0.4–34.4) *t* statistic 2.047, *p* value 0.0446. D/B Diff 22.5 (95%CI 8.4–36.6) *t* statistic 3.190, *p* value 0.0021. D/A Diff 23.0 (95%CI 9.1–36.6) *t* statistic 3.390, *p* value 0.0013

**Graph 3:**
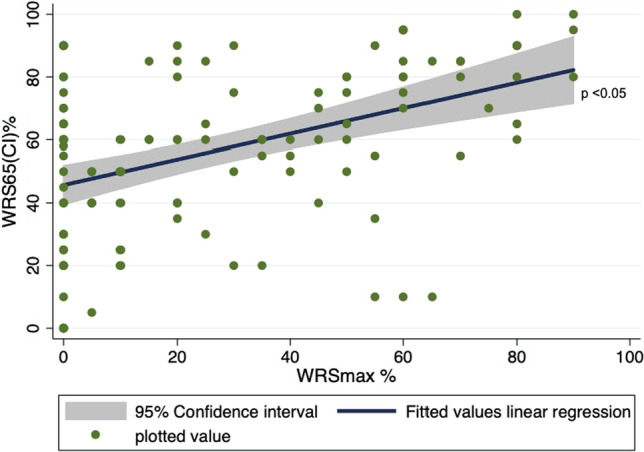
Fitted linear prediction plot showing association between pre-operative monosyllabic maximum word recognition score without hearing aid(WRSmax) and post-operative monosyllabic word recognition score at 65 dB (WRS65(CI)) after CI

**Graph 4:**
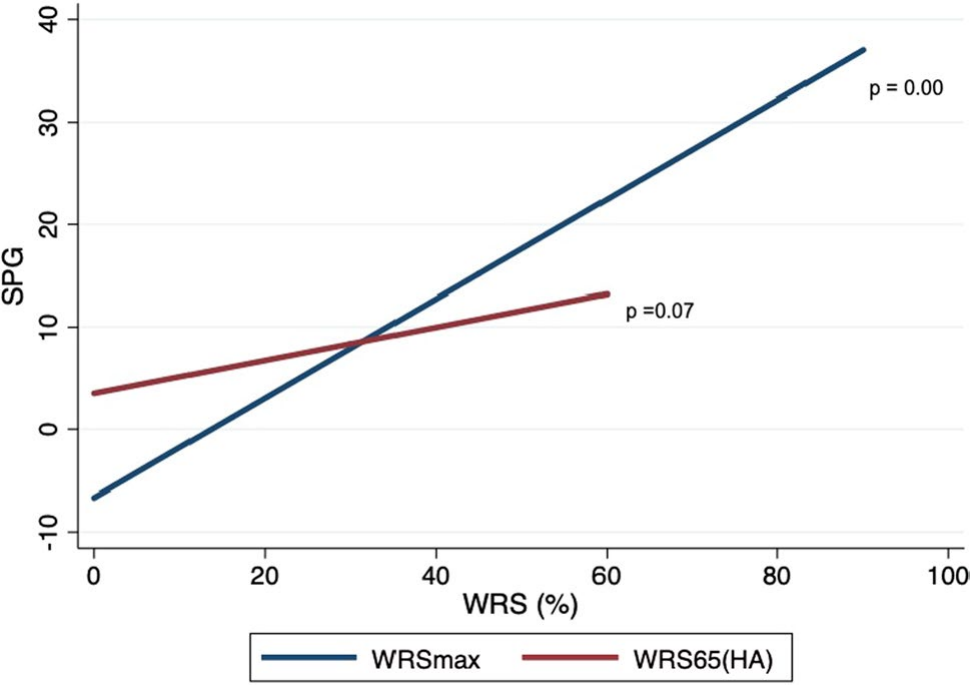
Fitted linear prediction plot showing association between pre-operative monosyllabic maximum word recognition score without hearing aid(WRSmax) and monosyllabic word recognition score with best fitted hearing aid at 65 dB (WRS65(HA)) with speech perception gap (SPG)

### Difference between ears with 0% WRSmax and more than 0% WRSmax

The size of the WRSmax0 group was 41 (32%) and WRSmax > 0 was found in 78 ears (61%). The mean WRS65(CI) for WRSmax0 was 47.3 ± 30% (median = 55%) showing varied results with a minimum of 0% and a maximum of 90%. In comparison the WRSmax > 0 group performed better with a mean WRS65(CI) of 61.3 ± 26% (median = 60%, min: 0%, max: 100%). Although the difference in median between the two groups was minimal, the difference in means between two groups was statistically significant (Difference 14 (95%CI 3.5–24.5) t statistic 2.646, P value 0.0093). Thus, in a normally distributed population, WRSmax > 0 group performed statistically significantly better with CI than WRSmax0 group.

### Association between various pre-operative audiological measurement and the outcome variable

The results of analysis of WRS65(CI) are shown in Table 3. Three variables (WRSmax > 0, WRS65(HA) and SPG) showed significant associations with WRS65(CI). After model fitting, a multivariate analysis involving these three variables showed collinearity and that WRS65(HA) was omitted. The final model showed a statistically significant association between WRS65(CI) and WRSmax > 0 (0.006, 95% CI 0.004–0.009, *p* value = 0.000) as shown in Graph 3, that is, as WRSmax > 0 increases, WRS65(CI) increased significantly. SPG was found to have an inverse association with WRS65(CI) in the final modeling, but wide statistical variation and relatively low statistical precision as evidenced by the confidence interval (− 0.003, 95% CI − 0.007 to − 1.80e−06, *p* value = 0.050).

**Table 3:** Univariate linear regression analyzing association between post-operative speech understanding after CI and various independent factors

Variables	Co efficient (95% CI)	*p* value
PTA4	− 0.002 (− 0.005 to 0.006)	0.121
WRSmax > 0	0.004 (0.002 to 0.006)	0.000
FNA4	− 0.002 (− 0.005 to 0.001)	0.226
WRS65(HA)	0.005 (0.003 to 0.007)	0.000
Speech perception gap	0.003 (0.0007 to 0.006)	0.013
Duration of hearing loss (in months)	0.00012 (− 0.0002 to 0.0003)	0.309
Duration of usage of hearing aids (in months)	0.002 (0.00002 to 0.0005)	0.061

### Speech perception gap

SPG was analyzed across two variables (WRSmax and WRS65(HA)). A positive linear association exists between SPG and WRSmax (0.5, 95% CI 0.41–0.56, *p* value = 0.000), but no associations were found between SPG and WRS65(HA) (0.2, 95% CI − 0.01 to 0.33, *p* value = 0.069) (Graph 4). Although there was no statistically significant association between SPG and WRS65(CI) in a linear regression, when categorized into groups, where in the SPG was ≥ 20% and < 20%, SPG ≥ 20% group performed much better post-operatively than the < 20% SPG group. The mean WRS65(CI) for the SPG ≥ 20% group was 63.8 ± 31% and the mean WRS65(CI) for SPG < 20% group was found to be 54.1 ± 26% (Chi square: − 9.7, *p* value = 0.07). To conclude as WRSmax increases, SPG increases significantly with SPG ≥ 20% group performing better than SPG < 20% group post-operatively.

### Role of duration of hearing loss and hearing aid usage

The mean UHL (*n* = 98) was 78.1 ± 152 months, while the median was found to be 0 showing that more than 50% of the ears had minimal UHL and, therefore, minimal auditory deprivation. There was no statistically significant association between UHL and WRS65(CI). UHL was not associated with SPG. After categorization, the number of ears with UHL0 was 58 and UHL > 0 was 40 (min:12, max 624, mean = 191 ± 188, median = 102 months). There was no difference between the mean WRS65(CI) for the two groups (UHL0: mean = 57.3 ± 29%, median = 60%; UHL > 0: mean = 56.3 ± 27%, median = 60%). Group UHL > 0 was statistically significantly inversely associated with WRSmax > 0 in the univariate linear regression (− 0.05, 95% CI: − 0.09 to − 0.01, *p* value = 0.011). Group UHL > 0 was not associated with CI65(HA) or with SPG. Thus, in patients with any time duration, wherein the hearing loss was unaided, WRSmax > 0 was significantly lower.

## Discussion

Among the 103 adults included in this study 21 patients had a moderate hearing loss on the contralateral side with the rest of the 82 patients having a severe to profound hearing loss. However, only 25 patients (30.5%) received a bilateral CI. This reflects the where a lot of adult patients who receive a CI are affected on both sides, but receive CI on only one side and continue to function with hearing aids on the opposite side, termed as bimodal hearing [2]. Although newer studies recommend bilateral CI as opposed to bimodal hearing rehabilitation, bilateral CI is not yet the standard as of now [25].

Especially in these cases, predicting outcomes after unilateral CI plays an important role in pre-operative counselling. The usual predictors quoted in literature are first, duration of deafness, where in more than 10 years of deafness is a negative factor, and second, pre-operative audiological scores and third, etiology of deafness [24]. In case of pre-operative audiological scores, even though it has been established in the recent years that speech understanding plays the most important role, the question of to what extent and which score is still debated. Even though pure tone audiogram is the first and most important step in identifying any level of hearing loss and is used unquestioningly to test hearing gain also when fitting hearing aids, tone hearing as such and in the form of hearing gain seems to play a very limited role in predicting post-operative outcomes. This is made clear across the two groups with better speech understanding (representing cochlear reserve) that perform better than the other two groups with worse speech understanding regardless of their tone hearing. It is again reinforced in the univariate regression analysis, wherein speech understanding in its various forms (WRSmax, WRS65(HA)) and SPG are associated with the outcome variable (WRS65(CI)). Thus, the better the pre-operative speech understanding which signifies the cochlear reserve of the patient, the better the post-operative outcome after CI. This is similar to the results of other studies [5, 12]. Holden et al. reported on 114 patients and found a correlation between preoperative sentence recognition score and post-operative monosyllabic score with Dowell et al. showing similar results in 300 patients. Another important idea that these results show us is, that patients with moderate hearing loss, as shown by tone hearing, but still suffer from bad speech perception, can definitely profit from CI [14]. This also demonstrates the need for testing of patients for speech understanding despite good tone hearing and the earlier start of hearing rehabilitation.

Among the various pre-operative speech understanding tests, even though WRS65(HA) is main the test used for CI indication, WRSmax plays a more important role in predicting the post-operative outcomes especially in post-lingually deaf adults [15]. WRSmax has been hypothesized to represent the information carrying capacity of the auditory system [11]. Very similar tone hearing as evidenced through pure tone audiograms most often lead to varying speech understanding levels, since tone hearing only shows the attenuation component of hearing loss. This shows the importance of measuring the maximum speech understanding that can be achieved under acoustic amplification when fitting hearing aids. The WRS65(HA) when combined with WRSmax tells us if the hearing aid is best fitted or if there is room for amplification. However, not all patients can convert their WRSmax into WRS65(HA) as shown in our study. Other studies have also recently reported that a considerable amount of hearing aid users, even with moderate hearing loss, were unable to convert their WRSmax into best fitted speech understanding at conversation levels [20, 21]. This mismatch has been attributed to insufficient dynamic range in these hearing aid users, combined with their intolerance of the high acoustic amplification [28]. This seriously limits the potential benefit of hearing aids in these patients. It also shows us that WRSmax better represents the information carrying capacity and the cochlear reserve of an ear than WRS65(HA) and thus better predicts the outcomes after CI as evidenced in the final linear regression model.

When discussing WRSmax in detail it should not be forgotten that there are always inherently two groups when considering CI: group with no reserve that is 0% WRSmax and a group with some reserve that is WRSmax above 0%. These groups have been reported to perform differently after CI; the cochlear reserve group understandably performing better than the group with no reserve [12, 15]. This was also evidenced in our study. This should, however, not discourage otologists from advising CI for the no reserve group. This group first has no other alternative for hearing rehabilitation, and second, this group shows extremely varied outcomes. Our study showed that the no reserve group with 0% WRSmax despite having a lesser mean outcome speech understanding than WRSmax > 0 group, was not far behind in terms of the median outcome speech understanding. With our cohort showing a minimal skewness, we struggle in clinically interpreting these results. That is whether to take the mean into consideration or the median in estimating the better performing group. While WRSmax is a good predictor of the outcome after CI of patients with some reserve (above 0% WRSmax), predicting post-operative outcomes for the no reserve group is not straightforward. The no reserve group thus remains an enigma when it comes to their post-operative outcomes.

When fitting hearing aids, patients are not routinely placed back in the testing booth to determine whether WRS65(HA) approaches WRSmax. Hearing aid providers typically operate under the assumption that the real-life hearing aid performance reaches the WRSmax. This is also because the recommendations tend to only include WRS65(HA) as an important means to verify the function of hearing aids [1, 8]. This is, however, not always true and many patients struggle to achieve their WRSmax levels with WRS65(HA). The difference between these two values gives the speech perception gap (SPG). This has been recently studied as an important factor, not only in fitting hearing aids, but also for recommending patients for earlier CI rehabilitation [6, 7]. This is because especially higher SPG implies that the hearing aids do not meet the cochlear reserve in these patients. These patients do tend to benefit from CI, as evidenced in our study, where the SPG ≥ 20% group performed better with 63.8 ± 31% mean WRS65(CI) than the SPG < 20% group with 54.1 ± 26% mean WRS65(CI). This result is clinically relevant and seems to suggest that rehabilitation with CI should not be delayed or deferred. Recent literature also suggests that in patients with SPG greater than 20% other technologies, such as middle ear hearing implants and/or CI may be more beneficial than traditional hearing aids [3, 20]. Patients with high WRSmax and SPG could also be considered for CI.

Even though the duration of hearing loss is an established factor affecting the outcome after CI, our study did not show such results. We attribute this to our small study population. The variable was collected from patient history and was perhaps not reliable. Another important factor in this study was the duration of hearing aid usage across the time the patients suffered from hearing loss. This could represent auditory deprivation and play a role in preserving the cochlear reserve and thus indirectly affect the outcome after CI. Our study measured this auditory deprivation as UHL. We found that even though there is no significant difference in outcome between minimal auditory deprivation group (UHL0) and the group with some to significant auditory deprivation (UHL > 0), the second group (UHL > 0) with a minimum of 12 months to a maximum of 624 months UHL was statistically significantly inversely associated with WRSmax > 0. This result should be interpreted with caution, since these data were also gathered from patient history and presents only an approximate value. Further research in this area is needed to determine the exact effect of the hearing aid usage on results after CI. However, early rehabilitation of patients with hearing aid who suffer from bad speech understanding regardless of tone hearing should still be considered.

As more and more patients with cochlear reserve are considered for hearing rehabilitation, superior hearing performance is the ultimate goal. Combining electrical stimulation through CI and acoustic amplification through hearing aids can enhance hearing performance. CI allows the damaged parts of the cochlea to be bypassed and provides electrical representation of the sound directly to the hearing nerve which is in turn interpreted by the brain. While the cues that are needed for speech understanding are usually extracted and preserved in patients receiving CI, full representation of the signal can still be limited. This is especially true for low frequency cues. While mid- and high frequency cues of consonant articulation are well conveyed by the CI, low frequency cues of voicing and fundamental frequency are conveyed poorly [23]. The quality of the signal can be improved by bimodal hearing, that is, CI on one ear and hearing aid on the other ear. Patients with bimodal hearing demonstrate improved hearing in background noise, improved sound quality, and improved satisfaction [16, 22]. This form of bimodal hearing was used in all 78 patients that received CI on one side in our cohort. Thus with widening indication criteria for CI, the potential for patients to benefit by continued use or addition of a hearing aid contralaterally should not be underestimated.

Another form of combining electric stimulation and acoustic amplification is through the use of hybrid implants [26]. Hybrid implants are typically indicated in patients with severe hearing loss in high frequencies with the presence of functional acoustic hearing at low frequencies (left corner curve) but continue to have poorer speech understanding due to their high frequency hearing loss. The group with relatively better speech understanding in our cohort did not fit these criteria. It is interesting to compare this group with possible hybrid candidates, but our cohort was not large enough to make meaningful comparisons.

### Limitations

In this study we have only looked at the role of tone and hearing and speech understanding in patients with CI. Other factors such as surgical techniques, device factors and implant models used haven been shown to affect post-operative performance after CI. These were not included in this study and this may limit the interpretation of the multivariate analysis. Relatively small sample size is another limitation of the study. We, therefore, included all patients in the analysis and the outliers (extremely good or bad performers after CI) were not analyzed separately. This might reduce the generalizability of the results.

## Conclusions

Cochlear reserve represented by WRSmax may play the most important role as a predictive factor of outcomes after CI and to a lesser extent WRS65(HA) and SPG. Tone hearing on the other hand seems to play a very minimal role, if any, in determining outcomes after CI. We suggest fitting hearing aids based on both WRSmax and WRS65(HA), with SPG being routinely measured. SPG may be considered when rehabilitating patients with good tone hearing and bad speech understanding. Further studies in exploring duration of usage of hearing aid in preserving cochlear reserve and thus improvement in optimal hearing aid usage are needed.
